# Circulating bone morphogenetic protein 8A is a novel biomarker to predict advanced liver fibrosis

**DOI:** 10.1186/s40364-023-00489-2

**Published:** 2023-04-27

**Authors:** Patricia Marañón, Stephania C. Isaza, Carlos Ernesto Fernández-García, Esther Rey, Rocío Gallego-Durán, Rocío Montero-Vallejo, Javier Rodríguez de Cía, Javier Ampuero, Ángela M. Valverde, Manuel Romero-Gómez, Carmelo García-Monzón, Águeda González-Rodríguez

**Affiliations:** 1 Metabolic Syndrome and Vascular Risk Laboratory, Hospital Universitario Santa Cristina, Instituto de Investigación Sanitaria del Hospital Universitario de La Princesa, Madrid, Spain; 2 SeLiver Group, Instituto de Biomedicina de Sevilla/CSIC/Hospital Virgen del Rocío, Seville, Spain; 3 Centro de Investigación Biomédica en Red de Enfermedades Hepáticas y Digestivas (CIBEREHD), Madrid, Spain; 4 Instituto de Investigaciones Biomédicas Alberto Sols (Centro Mixto CSIC-UAM), Madrid, Spain; 5 Centro de Investigación Biomédica en Red de Diabetes y Enfermedades Metabólicas Asociadas (CIBERDEM), Instituto de Investigaciones Biomédicas Alberto Sols (Centro Mixto CSIC-UAM), Madrid, Spain

**Keywords:** Liver fibrosis, Bone morphogenetic proteins, BMP8A, Non-invasive diagnosis, Hepatic stellate cells

## Abstract

**Background & Aims:**

Advanced hepatic fibrosis is the main risk factor of liver-related morbidity and mortality in patients with chronic liver disease. In this study, we assessed the potential role of bone morphogenetic protein 8A (BMP8A) as a novel target involved in liver fibrosis progression.

**Methods:**

Histological assessment and BMP8A expression were determined in different murine models of hepatic fibrosis. Furthermore, serum BMP8A was measured in mice with bile duct ligation (BDL), in 36 subjects with histologically normal liver (NL) and in 85 patients with biopsy-proven non-alcoholic steatohepatitis (NASH): 52 with non- or mild fibrosis (F0-F2) and 33 with advanced fibrosis (F3-F4). BMP8A expression and secretion was also determined in cultured human hepatocyte-derived (Huh7) and human hepatic stellate (LX2) cells stimulated with transforming growth factor ꞵ (TGFꞵ).

**Results:**

*Bmp8a* mRNA levels were significantly upregulated in livers from fibrotic mice compared to control animals. Notably, serum BMP8A levels were also elevated in BDL mice. In addition, in vitro experiments showed increased expression and secretion to the culture supernatant of BMP8A in both Huh7 and LX2 cells treated with TGFꞵ. Noteworthy, we found that serum BMP8A levels were significantly higher in NASH patients with advanced fibrosis than in those with non- or mild fibrosis. In fact, the AUROC of circulating BMP8A concentrations to identify patients with advanced fibrosis (F3-F4) was 0.74 (*p*˂0.0001). Moreover, we developed an algorithm based on serum BMP8A levels that showed an AUROC of 0.818 (*p*˂0.0001) to predict advanced fibrosis in NASH patients.

**Conclusion:**

This study provides experimental and clinical evidence indicating that BMP8A is a novel molecular target linked to liver fibrosis and introduces an efficient algorithm based on serum BMP8A levels to screen patients at risk for advanced hepatic fibrosis.

**Supplementary Information:**

The online version contains supplementary material available at 10.1186/s40364-023-00489-2.

## Introduction

Globally, chronic liver diseases (CLD) represent nearly 3.5% of the all-cause mortality and hepatocellular carcinoma (HCC) is the sixth leading cause of cancer [1], emphasizing the urgent need to implement multilevel interventions to tackle the CLD burden of health systems worldwide.

The underlying etiologies of CLD are multiple and include virus-induced chronic hepatitis, autoimmune biliary tract diseases, alcohol-related liver disease (ALD) and non-alcoholic fatty liver disease (NAFLD), largely its more progressive form named non-alcoholic steatohepatitis (NASH) which can occur in more than 40–50% of NAFLD-affected individuals [2]. CLD of any etiology are commonly featured by progressive hepatic fibrosis which, if untreated, can eventually lead to cirrhosis and/or HCC [3, 4].

Liver fibrosis is a dynamic process consisting in an increased production and deposition within the liver of extracellular matrix proteins (ECM), such as collagens among others, as a wound-healing response to chronic liver injury. Hepatic stellate cells (HSCs) are the main contributors to liver fibrosis when activated after a chronic damage and transdifferentiate from a quiescent phenotype into highly proliferative and profibrotic myofibroblasts which are the main ECM-producing cells [5].

It is well known that the stage of hepatic fibrosis is the major prognostic factor of liver-related morbidity and mortality in patients with CLD [6, 7] and, therefore, is often taken as a key data to choose the appropriate therapy and to establish the prognosis of a patient with CLD. Consequently, an accurate assessment of liver fibrosis is needed for successful clinical management of CLD patients. Liver biopsy is still considered the gold standard to differentiate hepatic fibrosis stages [8] but, given that it is an invasive and costly method with potential significant complications and sampling errors, non-invasive methods to assess hepatic fibrosis stages with accuracy are urgently needed. In this regard, transient elastography (TE), which uses pulsed-echo ultrasound as a surrogate marker of fibrosis, is now widely available and used in clinical practice, though it has some limitations derived from central obesity [9]. Therefore, the identification of non-invasive biomarkers of liver fibrosis has been gaining interest since they can offer a faster and cost-effective alternative to other diagnostic methods [10]. For instance, blood-based algorithms to predict liver fibrosis are mathematical models calculated from clinical or analytical data that have been validated for the quantification of the probability of advanced fibrosis state. Some of them, such as Fibrosis 4 score (FIB-4) and aminotransferase to platelet ratio index (APRI), have good sensitivity and specificity to rule out and rule in advanced fibrosis in CLD of varied etiologies [11, 12], but novel biomarkers or algorithms with higher accuracy to predict advanced fibrosis still remain as an unmet need.

Interestingly, a number of clinical and translational studies have shown that serum levels of different bone morphogenetic proteins (BMPs) are modulated in patients with CLD, particularly BMP2 [13], BMP7 [14–16], BMP9 and BMP10 [17]. As a member of BMP family, BMP8A has been described as developmental factor [18, 19]. In addition, circulating BMP8A has already been put forward as a novel biomarker for early diagnosis of thyroid cancer in humans [20], but little is known about its potential significance in CLD.

Taking this background into account, the aim of the present study was to determine the expression pattern of BMP8A in the liver of different preclinical models of hepatic fibrosis and to assess whether serum BMP8A levels could be a good predictor of liver fibrosis stage in patients with NASH, the commonest cause of CLD worldwide.

## Material and methods

### Fibrosis experimental models

C57BL6J mice were maintained at room temperature (22 °C) and controlled humidity, with light/dark cycles (12 h light/ 12 h dark), fed ad libitum and had free access to drinking water at the animal facilities of the Universidad Autónoma de Madrid (UAM). All animal experimentation was carried out following both Spanish and European legislations.

Animals were submitted to different experimental models in order to induce fibrosis. First, for the bile duct ligation (BDL) model, anaesthetized mice underwent BDL operation, which consisted in the double ligation with 4–0 silk ligatures and transection of the bile duct, as previously described [21]. Control animal group underwent a sham operation, where the common bile duct was exposed but not ligated (*N* = 7 animals per group). Mice were sacrificed 28 days after operation,

On the other hand, for the carbon tetrachloride (CCl_4_) treatment, mice were injected CCl_4_ (1:4 in olive oil, 1.6 mg/kg body weight) intraperitoneally (i.p.) twice-weekly while control mice were injected vehicle (olive oil) (*N* = 6 animals per group) and after 6 weeks of treatment mice were sacrificed.

Finally, for the non-alcoholic fatty liver disease (NAFLD) model, mice were fed either a high fat diet (D12492, Rodent Diet With 60 kcal% Fat Research Diets, Inc. New Brunswick, USA) or chow diet (CHD, SAFE A04-10 Panlab, Barcelona, Spain) for 16 weeks (*N* = 5 animals per group). At the end of each experiment, liver and serum samples were collected for further analysis.

### Study population

This study included 85 patients with biopsy-proven NASH, of them 52 showed non- or mild fibrosis (F0-F2) and 33 displayed advanced fibrosis stages (F3-F4). In addition, 36 subjects with histologically normal liver (NL) were also studied and considered as a control group. All patients met the inclusion criteria that included: age between 18 and 75 years old, less than 20 gr per day of alcohol consumption, absence of treatment with any potentially hepatotoxic drug, no analytical evidence of iron overload and seronegativity for HIV and hepatitis B and C infection.

This study was performed in agreement with the Declaration of Helsinki, and with local and national laws. The Human Ethics Committee of the Hospital Universitario Santa Cristina (report reference, PI-688A) and Hospital Universitario Virgen del Rocío (report reference, 0359-N-15) approved the study procedures, and all participants signed an informed written consent before inclusion in the study.

Clinical and biochemical characteristics of each patient were routinely determined by central laboratories. Blood-based fibrosis scores such as FIB-4 and APRI were calculated in the study population as previously described [11, 12]. Characteristics of all patients included in this study based on the histological diagnosis are shown in Table 1.

**Table 1:** Characteristics of the study population

	**Normal liver (NL) (*n* = 36)**	**Mild fibrosis (F0-F2) (*n* = 52)**	**Advanced fibrosis (F3-F4) (*n* = 33)**
Age (years)	56.82 ± 12.48	52.75 ± 11.89	60.64 ± 9.37^##^
Gender (female/male, %)	58.33 / 41.67	59.62 / 40.38	48.48 / 51.52
BMI (kg/m^2^)	27.72 ± 5.93	34.92 ± 7.99****	32.6 ± 4.63***
Glucose (mg/dL)	94.17 ± 12.76	125.9 ± 55.49**	120 ± 46.13**
Diabetes (%)	11.1	53.8****	75.8****^#^
Triglycerides (mg/dL)	107.2 ± 53.23	181 ± 133.7****	159.7 ± 74.75***
Total cholesterol (mg/dL)	186 ± 41.1	197.5 ± 46.83	194.4 ± 35.55
AST (IU/L)	19.5 ± 5.79	61 ± 121.6****	48.15 ± 29.74****
ALT (IU/L)	20.58 ± 12.03	67.33 ± 86.66****	54.97 ± 35.9****
GGT (IU/L)	40.39 ± 29.79	96.52 ± 158**	136.5 ± 189.1****^#^
Platelets (10^9^/L)	233.3 ± 65.5	234.1 ± 64.38	186.6 ± 68.56**^###^
Albumin (g/L)	44.85 ± 3.61	45.3 ± 3.64	44.71 ± 3.16
Fibrosis (%)			
Stage 0	100%	32.7%	
Stage 1		40.38%	
Stage 2		26.92%	
Stage 3			66.67%
Stage 4			33.33%

Data are shown as mean ± SD or as number of cases (%). BMI, body mass index; AST, aspartate aminotransferase

*ALT* Alanine aminotransferase, *GGT* Gamma-glutamyltransferase

^**^p < 0.01

^***^p < 0.005

^****^p < 0.0001, mild (F0-F2) or advanced fibrosis (F3-F4) vs. NL

^#^*p* < 0.05

^##^*p* < 0.01, advanced fibrosis (F3-F4) vs. mild fibrosis (F0-F2)

### Histopathology assessment

Paraffin embedded liver sections of 5 μm were stained with Haematoxylin and Eosin (H&E) and Sirius Red. Liver biopsy sections were examined by a single-blinded pathologist and evaluated according to the NAFLD activity score (NAS Score) system regarding presence of steatosis, inflammation and hepatocyte ballooning [22], as well as determination of the presence of fibrosis in four stages, being 0 absence of fibrosis and 4 cirrhosis [23]. Representative images were taken using an optical microscope Nikon Eclipse E400 (Nikon, Tokyo, Japan).

### Cell culture and treatment

Human hepatic stellate cells (HSC) LX2 (ATCC, Manassas, VA, USA) were maintained in a medium prepared with 50% Dulbecco´s modified Eagle´s medium (Ref. SH30022.01 DMEM, Cytiva, USA) containing high glucose and 50% DMEM containing high glucose without glutamine (Ref. 10,938–025 Gibco, Thermo Fisher Scientific, Inc.) and supplemented with antibiotics and 2% fetal bovine serum (FBS). LX2 cells were serum starved for 2 h prior to the treatment with TGFβ (Ref. 100-21C, Peprotech Inc., NJ, USA) (10 ng/ml) for 24 h. Human hepatoma cell line Huh7 (ATCC, Manassas, VA, USA) was maintained in DMEM containing high glucose, antibiotics and supplemented with 10% FBS. Huh7 cells were treated with TGFβ (10 ng/ml) for 24 h. All cells were cultured at 37 °C with 5% CO_2_ and relative humidity of 95%.

### Gene expression analysis

Total RNA was extracted from liver tissue samples or cell lysate using TRIzol reagent (Vitro, Sevilla, Spain) and was reverse transcribed with ImProm-II™ Reverse transcription kit (Promega Inc., Madison, WI, USA) in a T100TM Thermal Cycler (BioRad Inc., Madrid, Spain). Quantitative Real Time PCR (RTqPCR) was performed with StepOnePlusTM Real Time PCR System sequence detector (Thermo Fisher Scientific, Inc.) using SYBR Green method and quantified with ΔΔCt method. All samples were run in duplicate and normalized by *36B4*.

Primer sequences used were: m-*Bmp8a*, 5’ AACCATGCCATCTTGCAGTCT 3’ (forward) and 5’ CAGAGGTGGCACTCAGTTTGG 3’ (reverse); m-*Col1a1*, 5’ TAGGCCATTGTGTATGCAGC 3’ (forward) and 5’ ACATGTTCAGCTTTGTGGACC 3’ (reverse); m-*Acta2*, 5’ CCCAGACATCAGGGAGTAATGG 3’ (forward) and 5’ TCTATCGGATACTTCAGCGTCA 3’ (reverse); m-*Serpin1*, 5’ ATGACTGGGTGGAAAGGCATAC 3’ (forward) and 5’ CAGGCGTGTCAGCTCGTCTA 3’ (reverse); m-*36b4*, 5’ AGATGCAGCAGATCCGCAT 3’ (forward) and 5’ GTTCTTGCCATCAGCACC 3’ (reverse); h-*BMP8A*, 5’ CACCCTTCTCATCTGGATCG 3’ (forward) and 5’ CAGGAAGTAGGCACCGAGAG 3’ (reverse); h-*COL1A1*, 5’ ACTGGTGAGACCTGCGTGTA 3’ (forward) and 5’ GAATCCATCGGTCATGCTCT 3’ (reverse); h-*TGFβ1*, 5’ AGGACTGCGGATCTCTGTGT 3’ (forward) and 5’ GGGCAAAGGAATAGTGCAGA 3’ (reverse); h-*36B4*, 5’ CAGGCGTCCTCGTGGAAGTGAC 3’ (forward) and 5’ CCAGGTCGCCCTGTCTTCCCT 3’ (reverse).

### Determination of serum BMP8A levels

BMP8A concentration in serum samples was determined using the human BMP8A ELISA kit (CSB-EL002745HU, Cusabio Technology LLC, Hubei, China) for human samples and mouse BMP8A ELISA kit (MBS7205421, MyBioSource Inc., San Diego, CA, USA) for samples from mice, following manufacturer’s indications. Absorbance from samples was interpolated to a standard curve using a four-parameter logistic (4-PL) equation.

### Protein content analysis by western blot

After treatment with TGFβ, LX2 and Huh7 cells were scraped in RIPA buffer (50 mM Tris HCl, pH 7.4, 1% Triton X-100, 0.2% SDS, 1 mM EDTA, 1 mM PMSF and 5 μg/ml leupeptin) to obtain total protein extracts. Also, protein content from LX2 and Huh7 culture supernatants were concentrated using ultrafiltration units (Vivaspin Turbo 4 Ultrafiltration Unit.VS04T21, Sartorius, Thermo Fisher Scientific Inc.). Protein extracts and concentrated supernatants were boiled in Laemmli sample buffer prior to electrophoresis in 10% SDS-PAGE. Proteins were then transferred to an immunoblot nitrocellulose membrane (Bio-Rad) that was blocked with 5% non-fat dry milk and exposed to primary BMP8A antibody (ab154373 Abcam plc, Cambridge, UK), αSMA (A-2547, Merck Life Science, Darmstadt, Germany) and COL1A1 (SC-8784, Santa Cruz Biotechnology Inc., Heidelberg, Germany) antibodies overnight at 4 °C. After the incubation with the corresponding secondary antibody (Santa Cruz Biotechnology Inc.), immunoreactive bands were visualized using the ECL Western blotting protocol (Bio-Rad). Densitometric analysis of the bands was performed using Image J software. Ponceau staining was used as loading control.

### Statistical analysis

Kolmogorov–Smirnov test was applied to evaluate if the variables were adjusted or not to a normal distribution. Qualitative variables are presented as relative frequencies, and data between groups compared with Pearson’s Chi-squared or Fisher’s exact test as appropriated. Quantitative variables are expressed as measures of central tendency (mean) and dispersion (standard deviation -SD- for patients’ data or standard error of mean -SEM- for experimental data). Data between groups were compared with Student´s t test for variables following a normal distribution and Mann–Whitney U test for continuous variables following a non-parametric distribution. Univariate and multivariate binary logistic regression analysis adjusted for confounders were used to test the independence of the associations of dependent variable (fibrosis stage) with those significant in univariate models and with those considered of clinical relevance. In addition, multivariate regression models were constructed and parameters selected by stepwise method based on likelihood ratio test. Box-Tidwell procedure was used for testing linearity of logit, and the goodness of fit of the model was evaluated using the Hosmer–Lemeshow statistic. In order to assess diagnostic accuracy of distinct regression models constructed, receiver operating characteristics (ROC) curves and area under the ROC curve (AUROC) were carried out. All statistical analyses were performed using the GraphPad Prism 6.0 software (GraphPad Software Inc., San Diego, CA, USA) and the IBM SPSS Statistics 24.0 (SPSS Inc., IBM, Armonk, NY) software with two-sided tests, with a *p* value of < 0.05 considered as statistically significant.

## Results

### Increased hepatic BMP8A expression in fibrosis experimental models

**Fig. 1:**
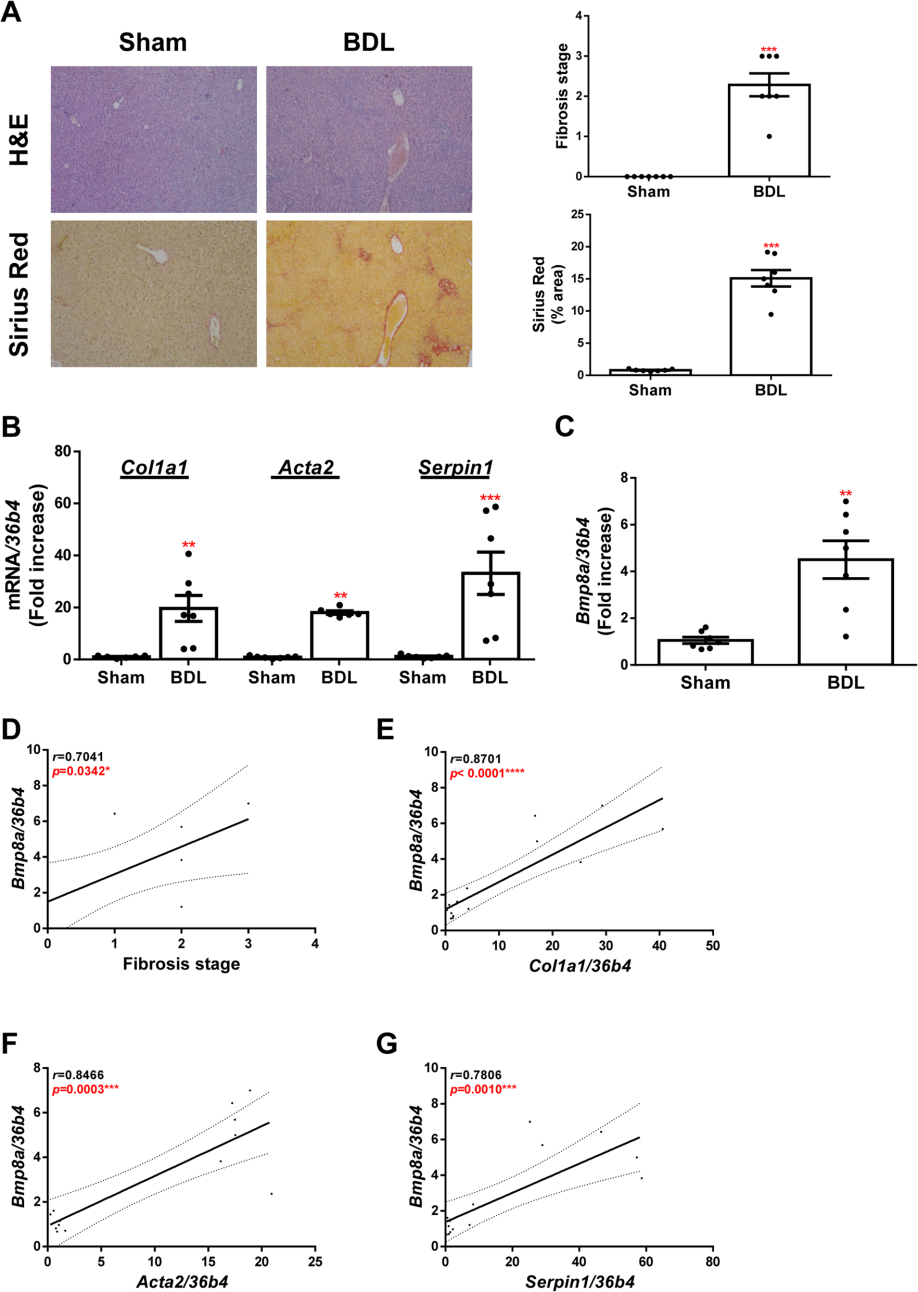
Increased hepatic *Bmp8a* expression in BDL mice. **A** Representative images of H&E and Sirius Red staining. Evaluation of the fibrosis stage (upper panel) and quantification of Sirius Red expressed as % of stained area (lower panel). **B** Hepatic mRNA levels of *Col1a1, Acta2* and *Serpin1* determined by RT-qPCR and normalized to *36b4* gene expression. **C** Hepatic mRNA levels of *Bmp8a* determined by RT-qPCR and normalized to *36b4* gene expression. **D**, **E**, **F** and **G** Correlation of matched *Bmp8a* mRNA expression with fibrosis stage, *Col1a1, Acta2* and *Serpin1* mRNA expression, respectively. Experimental conditions: mice with either BDL or sham operation and sacrificed 28 days after operation (*N* = 7 animals per group). BDL, bile duct ligation. Data are expressed as fold increase relative to control condition (sham group, 1) and presented as mean ± SEM. *p < 0.05, **p < 0.01, ***p < 0.005 and ****p < 0.0001, BDL *vs.* Sham

Firstly, to determine BMP8A expression during liver fibrosis, we established a BDL-induced liver fibrosis model in mice. Upon histological examination at 28 days after BDL, mice exhibited a large area of collagen deposition, assessed by Sirius Red staining, compared to the control group (Fig. 1A). As expected, mRNA expression of important modulators of the hepatic fibrogenic process, including collagen type 1 (COL1A1, *Col1a1*) and α-smooth muscle actin (αSMA, *Acta2*), was induced in livers of BDL mice, in parallel with a well-characterized fibrotic marker*,* plasminogen activator inhibitor type 1, (PAI1, *Serpin1*) (Fig. 1B). Furthermore, hepatic BMP8A expression was also significantly upregulated in BDL mice (Fig. 1C), showing a positive correlation between hepatic mRNA levels of BMP8A and the fibrosis stage (Fig. 1D) as well as with the mRNA levels of fibrotic markers analyzed (Fig. 1E-G).

In order to determine whether elevated BMP8A expression observed in the liver of BDL mice was due to the fibrogenic process and not to the cholestasic liver injury induced by BDL, we determined its hepatic expression profile in other experimental fibrosis models. For that goal, mice received intraperitoneal injections of CCl_4_, a classic preclinical model for liver fibrosis induction, and were sacrificed after 6 weeks. The quantitative analysis showed that mRNA levels of both COL1A1 and PAI1 were markedly increased (Figure S1A) in parallel to *Bmp8a* mRNA levels observed in livers of CCl_4_-treated mice (Figure S1B-S1D).

**Fig. 2:**
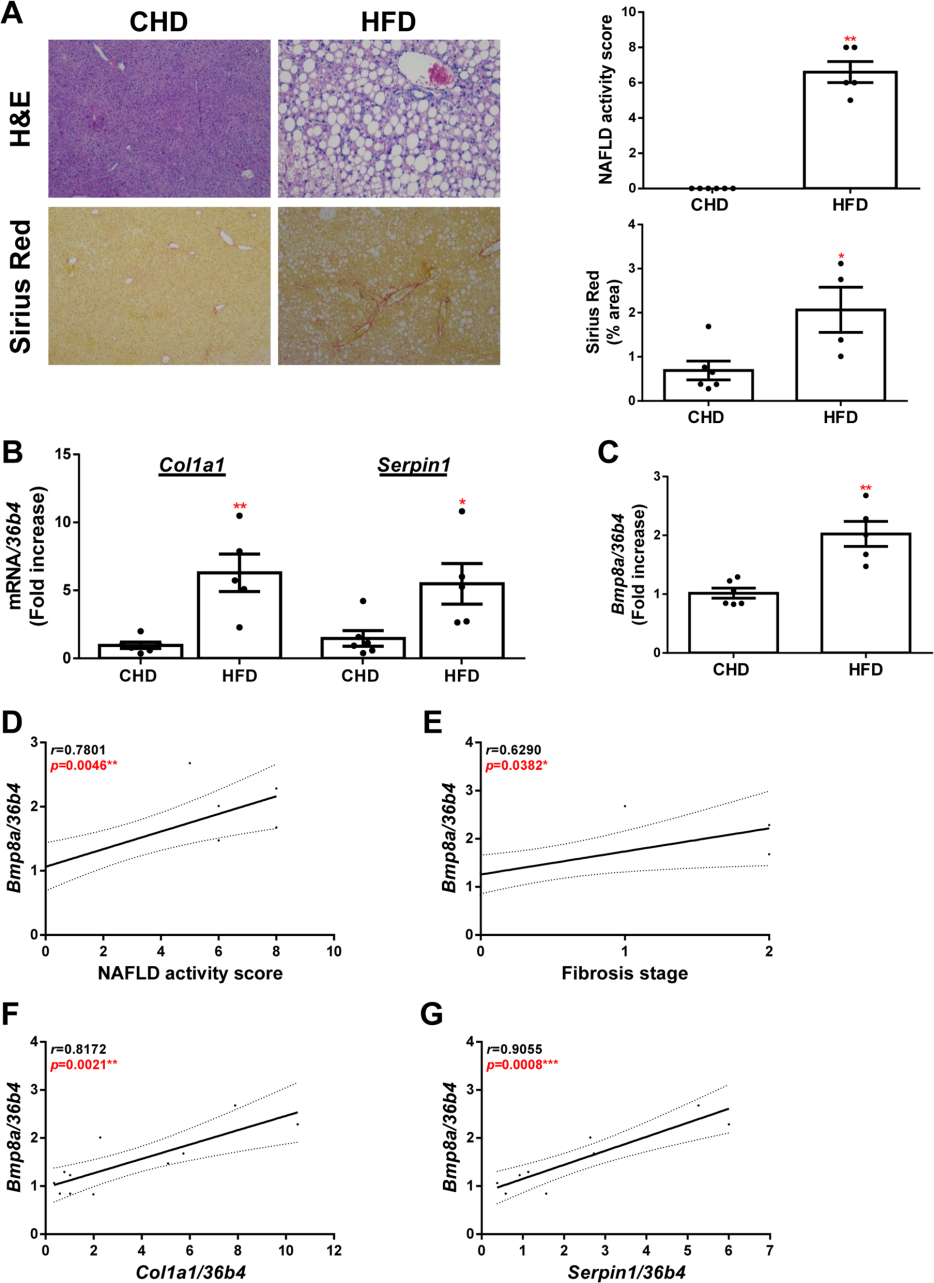
Hepatic *Bmp8a* expression is upregulated in NAFLD mice. **A** Representative images of H&E and Sirius Red staining. NAFLD activity score evaluation (upper panel) and quantification of Sirius Red expressed as % of stained area (lower panel). **B** Hepatic mRNA levels of *Col1a1* and *Serpin1* determined by RT-qPCR and normalized to *36b4* gene expression. **C** Hepatic mRNA levels of *Bmp8a* determined by RT-qPCR and normalized to *36b4* gene expression. **D**, **E**, **F** and **G** Correlation of matched *Bmp8a* mRNA expression with NAFLD activity score, fibrosis stage, *Col1a1* and *Serpin1* mRNA expression, respectively. Experimental conditions: mice fed with HFD or CHD (control group) for 16 weeks (*N* = 5 animals per group). NAFLD, nonalcoholic fatty liver disease. Data are expressed as fold increase relative to control condition (CHD group, 1) and presented as mean ± SEM. *p < 0.05, **p < 0.01 and ***p < 0.005, HFD *vs.* CHD

Finally, we performed a preclinical model of NAFLD since liver fibrosis can appear during NASH progression. Mice were fed a regular chow diet (CHD group) or high-fat diet (HFD group) for 16 weeks. As expected, livers of HFD mice displayed NASH features, such as steatosis, lobular inflammation and ballooning (Fig. 2A). Hepatic fibrosis, evaluated by Sirius Red staining (Fig. 2A), and induction of fibrogenic markers (Fig. 2B) were only observed in mice fed with HFD. We then found that *Bmp8a* gene expression was significantly increased in HFD fed mice with respect to control animals (Fig. 2C). Noteworthy, we found a significant positive correlation between the hepatic *Bmp8a* mRNA levels and the NAFLD activity score and the stage of fibrosis (Fig. 2D-E), and between the hepatic mRNA levels of *Bmp8a* and different fibrotic markers (Fig. 2F-G).

### BMP8A expression and secretion in human liver cells

In order to clarify which liver cells can be the cellular source of BMP8A, we performed some in vitro experiments in an attempt to reproduce the experimental conditions of the murine models. To that end, BMP8A levels were determined in both LX2 and Huh7 cells stimulated with TGFβ, a crucial mediator of fibrogenesis in liver diseases [24].

**Fig. 3:**
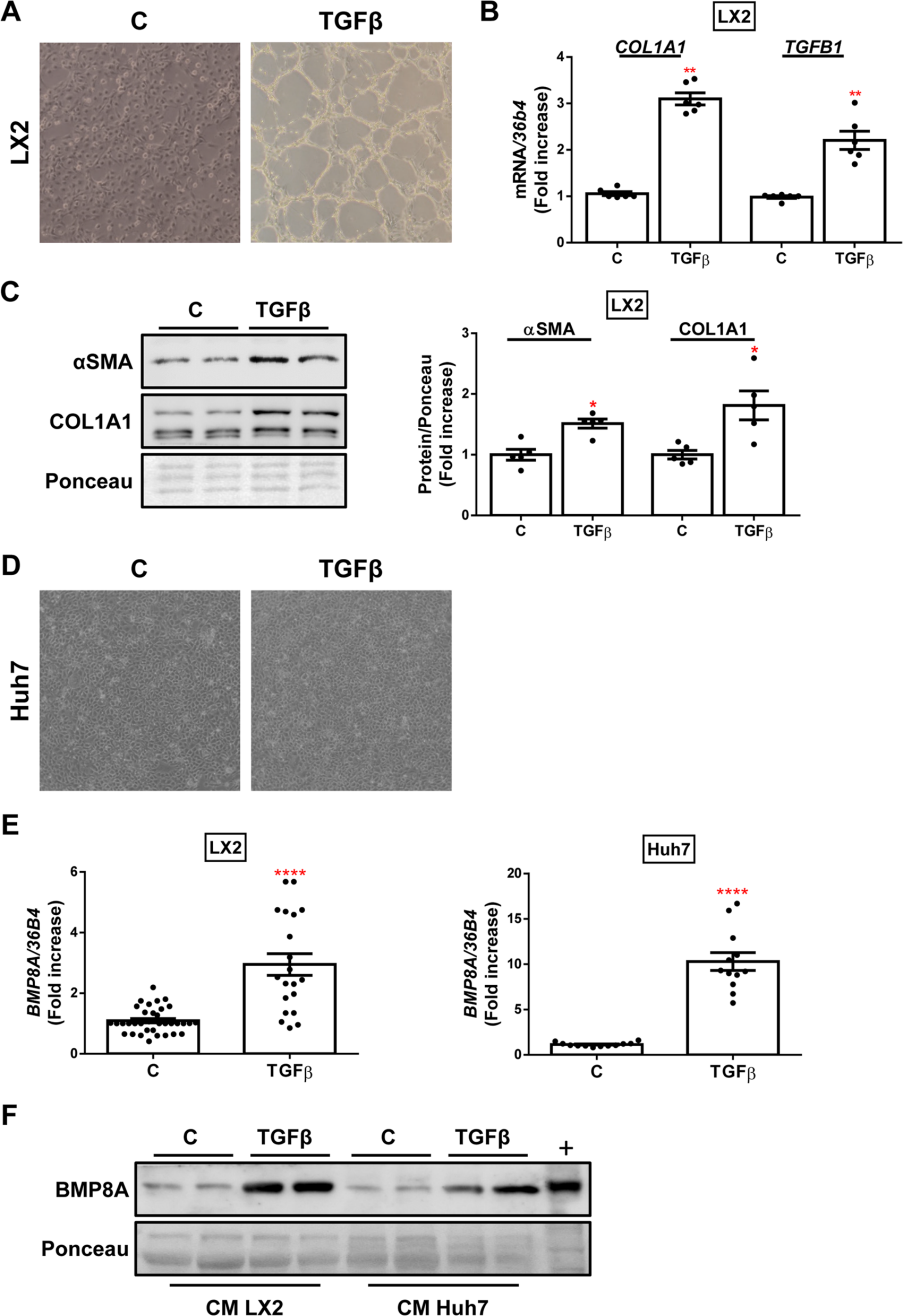
TGFβ induces BMP8A expression and secretion in LX2 HSCs and in Huh7 cells. **A** Representative phase-contrast images. **B** mRNA levels of *COL1A1 and TGFB1* determined by RT-qPCR and normalized to *36B4* gene expression. **C** Representative blot of cell lysates with αSMA and COL1A1 antibodies. Ponceau staining as loading control. **D** Representative phase-contrast images. **E** mRNA levels of *BMP8a* determined by RT-qPCR and normalized to *36B4* gene expression. **F** Representative blot of CM with BMP8A antibody. Ponceau staining as loading control. As positive control ( +), HEK293 whole cell lysate was used. Experimental conditions: LX2 or Huh7 cells treated with TGFβ 10 ng/ml for 24 h (*n* ≥ 3 independent experiments performed by duplicate). Huh7, human hepatocyte-derived cell line. LX2, human HSC line. CM, cultured media. Data are expressed as fold increase relative to control condition (C, 1) and presented as mean ± SEM. *p < 0.05, **p < 0.01 and ****p < 0.0001 TGFβ *vs.* C

Once HSC activation was achieved by TGFβ treatment in LX2 cells, as assessed by the change of morphological features (Fig. 3A) and the induction of fibrogenic markers (Fig. 3B and 3C), *BMP8A* expression was measured. In parallel, gene expression levels of this BMP protein were also analyzed in Huh7 cells after TGFβ treatment. Noteworthy, hepatocyte morphology did not change significantly under any experimental condition (Fig. 3D). Significantly higher *BMP8A* mRNA levels were observed in both LX2 and Huh7 cells treated with TGFβ (Fig. 3E). Interestingly, a higher BMP8A protein amount was also found in the supernatant of TGFβ-treated cells (Fig. 3F). These results indicate that TGFβ is sufficient to induce BMP8A expression and secretion in HSCs and hepatocytes.

### Association of serum BMP8A levels with liver fibrosis

**Fig. 4:**
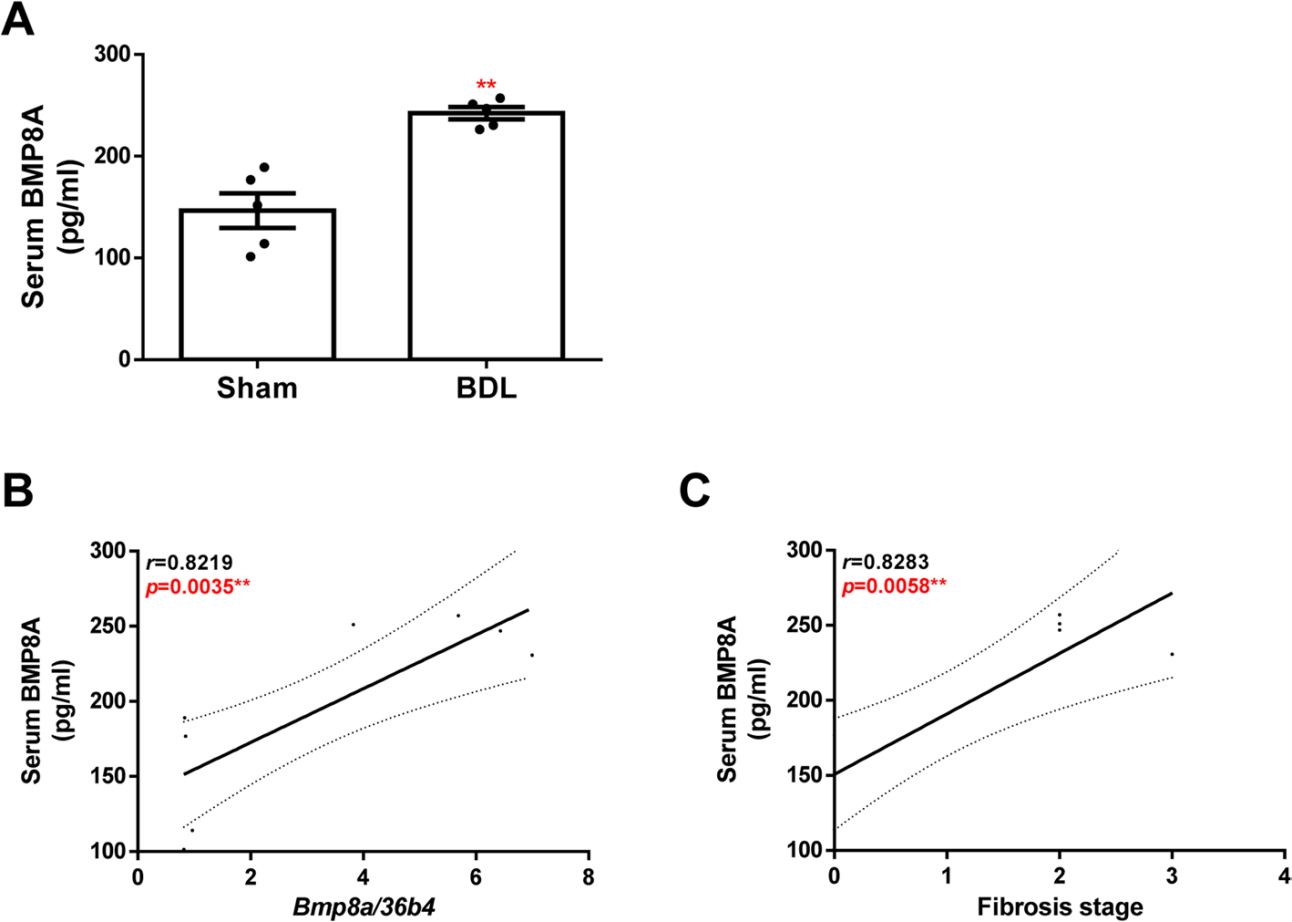
Circulating BMP8A levels are increased in BDL mice. **A** Serum levels of BMP8A determined by ELISA. Data are expressed as pg/ml and presented as mean ± SEM. **B** and **C** Correlation of matched serum BMP8A levels with hepatic *Bmp8a* mRNA expression and fibrosis stage, respectively. Experimental conditions: mice with either BDL or sham operation and sacrificed 28 days after operation (*N* = 7 animals per group). BDL, bile duct ligation. **p < 0.01, BDL *vs.* Sham

As the treatment of hepatic cells with TGFβ induced the release of BMP8A to the supernatant, we wondered whether the increase of hepatic *Bmp8a* expression found in livers from fibrotic mice might be reflected in the circulation. Thus, an assay for BMP8A detection of serum samples from mice was performed. Serum BMP8A levels were higher in BDL mice compared to control animals (Fig. 4A), and a statistically significant association was found between serum BMP8A levels and its hepatic expression pattern and with the hepatic fibrosis stage as well (Fig. 4B-C). These data suggest that BMP8A might be a useful biomarker of liver fibrosis.

### BMP8A is elevated in serum from patients with liver fibrosis

**Fig. 5:**
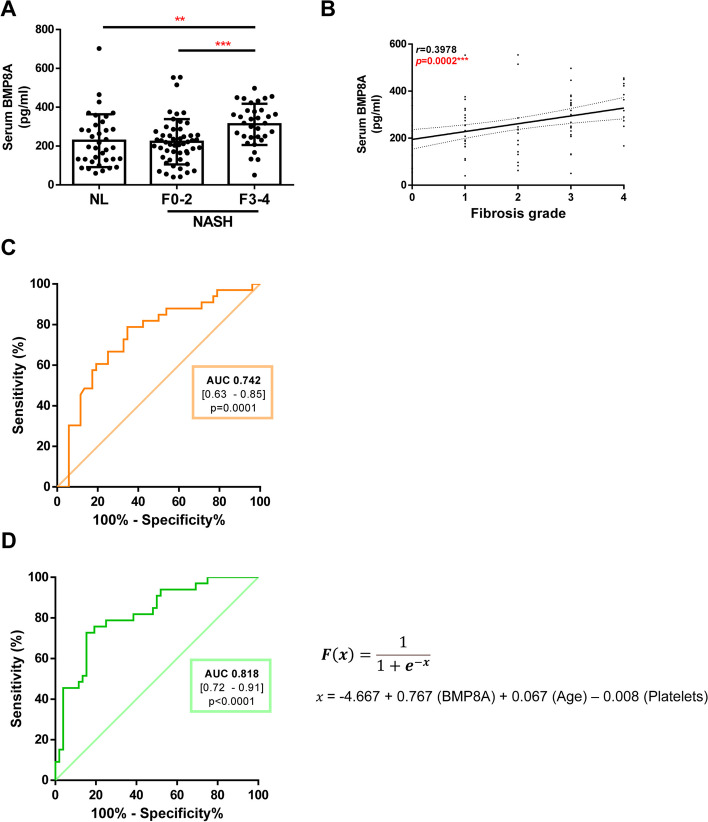
Increased serum BMP8A levels in NASH patients with advanced fibrosis. **A** Serum levels of BMP8A determined by ELISA. Data are expressed as pg/ml and presented as mean ± SD. **B** Correlation in the study population of matched serum BMP8A levels with fibrosis stage. **C** AUROC of BMP8A univariate analysis to predict advanced fibrosis (F3-F4). **D** AUROC of multivariate analysis. Study population: 85 NASH patients, 52 with non or mild fibrosis (F0-F2) and 33 with advanced fibrosis (F3-F4), and 36 subjects with normal liver. NASH, nonalcoholic steatohepatitis. AUROC, area under the ROC curve. *p < 0.05, **p < 0.01 and ***p < 0.005, F3-4 vs. F0-2 or NL

Taking into account the results from the experimental models which indicate a potential relevance of serum BMP8A levels for predicting the fibrosis stage, we measured circulating BMP8A levels in NASH patients with and without advanced hepatic fibrosis as well as in subjects with histologically normal liver (NL). Among patients with NASH (*N* = 85), circulating BMP8A concentrations were significantly increased in patients with advanced fibrosis (F3-F4) (312.1 ± 106.5 pg/mL, *N* = 33) compared to those without or with mild fibrosis (F0-F2) (222.3 ± 116.8 pg/mL, *p* = 0.0001, *N* = 52), as well as compared to individuals with NL (227.8 ± 136 pg/mL, p = 0.0019, *N* = 36, Fig. 5A). Notably, no significant difference in serum BMP8A levels was found between NASH patients with F0-F2 and individuals with NL. In addition, a significant and progressive elevation in serum BMP8A levels was found in NASH patients following the increase of hepatic injury towards significant fibrosis stages (*r* = 0.3978, *N* = 85, *p* = 0.0002, Fig. 5B).

### BMP8A is an independent predictor of advanced liver fibrosis

Finally, in order to evaluate the usefulness of serum BMP8A for predicting advanced liver fibrosis (F3-F4) in NAFLD patients, the area under the ROC curve (AUROC) was analyzed in the entire study population. The AUROC of circulating BMP8A concentrations to identify patients with advanced fibrosis (F3-F4) was 0.74 (CI 95% (0.63–0.85); *p* ˂ 0.0001) (Fig. 5C). Since direct and indirect biomarkers may be used alone or, more commonly, in combination with other clinical variables to develop composite scores, we combined serum BMP8A levels with other clinically relevant independent variables to perform multivariate logistic regression analyses in the whole study population (Table 2). Circulating BMP8A was identified as an independent predictor of advanced fibrosis (OR = 2.15 for each 100 pg/mL, *p* = 0.002) together with age (OR = 1.07, *p* = 0.02) and platelet count (OR = 0.99, *p* = 0.07). Based on this model, we developed the following algorithm called BMP8A fibrosis score (BFS) which had excellent performance (AUROC = 0.818, CI 95% (0.72–0.91), *p*˂0.0001) (Fig. 5D). Diagnostic accuracy of the model was evaluated through ROC analysis at different cut off points for the predicted probability of the model, which revealed good overall performance (Table 3). Moreover, Youden Index set the best cut-off point at 0.46, which was adequate to rule in advanced fibrosis with a specificity of 84.6% and to rule out advanced fibrosis with a sensitivity of 69.7%. The PPV and NPV were 74.2% and 81.5%, respectively. In fact, comparing its accuracy with other validated scores for fibrosis diagnosis, BFS showed better AUROC curve value than APRI or FIB-4 (0.651, CI 95% (0.53–0.77) and 0.756, CI 95% (0.65–0.86) respectively) in our study population. Hence, with a single cut-off point set at 0.46, BFS had better overall accuracy (78.8%) than APRI or FIB-4 to rule in (60.0% and 65.9%, respectively) and to rule out (62.3% and 64.7%, respectively) (Table 4). On one hand, BFS showed higher PPV than APRI and FIB-4 to rule in (74.2% vs. 44.4% and 64.3%, respectively) and better positive likelihood ratio (4.5 vs. 1.3 and 2.8, respectively). On the other hand, to rule out, NPV was better in BFS than in both APRI and FIB-4 scores (81.5% vs. 71.7% and 80.6%, respectively), while negative likelihood ratio was similar to FIB-4 (0.4) and better than APRI (0.6). These results suggest that BFS performance was better than APRI or FIB4 to rule in and to rule out advanced fibrosis in our study population.

**Table 2:** Univariate and multivariate analysis of the independent variables associated with advanced fibrosis in the study population

	**Univariate analysis**			**Multivariate analysis**		
**Independent variables**	**OR**	**95% CI**	***p* value**	**OR**	**95% CI**	***p* value**
BMP8A (100 pg/ml)	2.01	[1.3–3.1]	0.002	2.15	[1.33–3.48]	0.002
Sex (female/male)	1.56	[0.65–3.78]	0.316			
Age (years)	1.07	[1.02–1.13]	0.004	1.07	[1.01–1.13]	0.02
BMI (kg/m^2^)	0.95	[0.88–1.02]	0.308			
ALT (IU/L)	0.99	[0.98–1]	0.464			
AST (IU/L)	0.99	[0.99–1]	0.574			
GGT (IU/L)	1	[0.99–1]	0.309			
Platelets (10^9^/L)	0.99	[0.98–1]	0.004	0.99	[0.98–1]	0.07
Albumin (g/L)	0.95	[0.82–1.09]	0.484			

*OR* Odds ratio, *CI* Confidence interval, *BMI* Body mass index, *AST* Aspartate aminotransferase, *ALT* Alanine aminotransferase, *GGT* Gamma-glutamyltransferase

**Table 3:** Diagnostic accuracy of BFS

BFS cutoff point	Accuracy	%	SN	SP	PPV	NPV	LR +	LR-
≥ 0.10	54.1	82.4	97.0	26.9	45.7	93.9	1.3	0.1
≥ 0.20	62.4	71.8	93.9	42.3	50.8	91.7	1.6	0.1
≥ 0.30	70.6	51.8	78.8	65.4	59.1	82.9	2.3	0.3
≥ 0.40	76.5	43.5	75.8	76.9	67.6	83.3	3.3	0.3
≥ 0.46	**78.8**	**36.5**	**69.7**	**84.6**	**74.2**	**81.5**	**4.5**	**0.4**
≥ 0.50	77.6	35.3	66.7	84.6	73.3	80.0	4.3	0.4
≥ 0.60	72.9	25.9	48.5	88.5	72.7	73.0	4.2	0.6
≥ 0.70	70.6	14.1	30.3	96.2	83.3	68.5	8.0	0.7
≥ 0.80	65.9	9.4	18.2	96.2	75.0	64.9	4.8	0.9
≥ 0.90	62.3	1.2	3.0	100	100	61.9	-	1.0

*BFS* BMP8A fibrosis score, *%* number of patients with BFS ≥ cut-off point, *SN* Sensitivity, *SP* Specificity, *PPV* Positive predictive value, *NPV* Negative predictive value, *LR* + positive likelihood ratio, *LR-* Negative likelihood ratio

**Table 4:** Comparison of the operating characteristics of BFS and other widely used fibrosis scores to detect high risk of advanced fibrosis (F3-F4)

	**BFS**	**APRI**		**FIB-4**	
Operating characteristics	**≥ 0.46**	**< 0.5**	**≥ 1.5**	**< 1.30**	**≥ 2.67**
Accuracy	78.8	62.3	60.0	64.7	65.9
%	36.5	54.1	10.6	42.4	16.5
SN	69.7	60.6	12.1	78.8	27.3
SP	84.6	63.5	90.4	55.8	90.4
PPV	74.2	51.3	44.4	53.1	64.3
NPV	81.5	71.7	61.8	80.6	66.2
LR +	4.5	1.7	1.3	1.8	2.8
LR-	0.4	0.6	1.0	0.4	0.8

*BFS* BMP8A fibrosis score, *%* number of patients with BFS ≥ cut-point, *SN* Sensitivity, *SP* Specificity, *PPV* Positive predictive value, *NPV* Negative predictive value, *LR* + Positive likelihood ratio, *LR-* Negative likelihood ratio

## Discussion

In the present study we provide data revealing, for the first time, that hepatic *Bmp8a* expression is upregulated in distinct preclinical models of liver fibrosis and positively correlated with the stage of liver fibrosis, as well as with the hepatic mRNA levels of different markers of fibrogenesis. In addition, we identified that TGFβ, a key mediator of liver fibrogenesis, induces BMP8A expression and secretion in both HSCs and hepatocytes.

Previous experimental studies have reported that different members of the BMP family are elevated in fibrotic livers. In that regard, Zhang et al. observed that BDL rats showed increased hepatic BMP7 expression compared with control group [25]. Moreover, BMP7 protein levels were upregulated in the livers of acute CCl_4_-injured mice as well, but not in the livers from mice submitted to a chronic CCl_4_ challenge [26]. Notably, an enhanced hepatic BMP6 expression was only detected in diet-induced NAFLD mice but not in both BDL and CCl_4_ models of liver fibrosis [27]. In line with our results, hepatic BMP8B levels were upregulated in both diet-induced NASH and chronic CCl_4_ mouse models in parallel to the fibrosis stage [28, 29]. On the contrary, hepatic *Bmp9* mRNA and protein expression was downregulated in a cholestatic mouse model [30]. These studies, along with our results, indicate that BMPs might play an important role in liver fibrosis pathogenesis. Of relevance, our findings herein suggest that BMP8A could be an important mediator of liver fibrosis progression, regardless the etiology of chronic liver damage; however, further experimental studies are needed to elucidate the precise role of BMP8A in the pathophysiology of liver fibrogenesis.

One of the most striking findings of this study was that the increased hepatic *Bmp8a* expression in BDL mice was reflected in elevated serum levels of this BMP, which also correlated with the hepatic fibrosis stage, positioning this protein as a potential biomarker for non-invasive detection of advanced liver fibrosis. Taking this into account, we measured BMP8A in serum from NASH patients with or without advanced fibrosis as well as in individuals with histologically normal liver (NL). Notably, serum BMP8A levels were significantly higher in NASH patients with advanced fibrosis (F3-F4) not only than in subjects with NL but, what it is even more interesting, than in NASH patients without or with mild fibrosis (F0-F2).

It is noteworthy to highlight that this is the first study measuring serum BMP8A concentrations in patients with liver fibrosis. Other studies have previously reported that serum levels of distinct BMPs can be detected in patients with CLD. In this regard, we have just reported that serum levels of BMP2 were significantly elevated in NAFLD patients with respect to subjects with histologically NL [13]. Furthermore, it has been shown that circulating BMP7 levels were increased in patients with CLD of different etiologies [14, 15, 31]. Conversely, serum levels of both BMP9 and BMP10 were decreased in patients with cirrhosis compared to patients with pre-cirrhotic liver fibrosis, being associated with disease severity [17]; and a decreased concentration of circulating BMP9 was observed in patients with NAFLD [32].

Another relevant finding to point out in the present study is that serum BMP8A levels are significantly associated with the hepatic fibrosis stage of the NASH patients studied, indicating that circulating BMP8A may be a useful biomarker to distinguish advanced hepatic fibrosis from mild fibrosis. Accordingly, on the univariate logistic regression analysis, serum BMP8A levels were significantly associated with advanced fibrosis with a good accuracy to identify NASH patients with F3-F4 stages (AUROC, 0.742). In order to improve the accuracy of advanced fibrosis detection, we combined serum BMP8A levels with other clinically relevant variables and, based on the formula from multivariate logistic regression model, we developed an algorithm called BMP8A fibrosis score (BFS) combining circulating BMP8A (OR = 2.15 for each 100 pg/mL, *p* = 0.002) with age (OR = 1.07, *p* = 0.02) and platelet count (OR = 0.99, *p* = 0.07) which had an excellent performance to predict advanced fibrosis in our NASH patients (AUROC = 0.818, *p* ˂ 0.0001).

It is important to mention that the novel blood-based algorithm described herein and named BFS performed better than both APRI and FIB-4 index, two of the most widespread scoring systems to evaluate liver fibrosis in patients with CLD of different etiologies [11, 12], to predict advanced hepatic fibrosis in the study population. Currently, most of the fibrosis scores use two different cut-off points to rule in and to rule out advanced fibrosis, consequently establishing a grey zone with a significant proportion of unclassified patients. According to APRI and FIB-4 categorization, the grey zone represented 35.3% and 41.1% of patients in our study population, respectively. The strength of BFS relies on its capacity to accurately distinguish mild from advanced fibrosis using a single cut-off point, thus avoiding unclassified patients, and most importantly, without heavily sacrificing sensitivity or specificity.

The main limitation of our study is that we have included only NASH patients with liver fibrosis and, therefore, the results might be different in patients with CLD of different etiologies. However, our preclinical studies showed that BMP8A expression was upregulated in different experimental models of liver fibrosis. Further studies in distinct and larger cohorts of patients with CLD of varied etiologies are needed to validate the potential utility in clinical practice of BFS as a non-invasive algorithm to identify/screen those CLD patients at high-risk of advanced fibrosis.

In conclusion, the results of the present study provide the first scientific evidence that hepatic and serum levels of BMP8A are abnormally elevated in experimental models of liver fibrosis and in the serum of NASH patients with advanced hepatic fibrosis, indicating that BMP8A is a new molecular target linked to liver fibrogenesis. In addition, we have defined a simple and efficient algorithm termed BFS which was able to discriminate advanced hepatic fibrosis with a good accuracy in NASH patients. Validation of these results in larger and independent cohorts of CLD patients are warranted to propose BFS as a clinically useful tool to screen CLD patients at primary care settings, in order to identify those patients at higher risk of advanced fibrosis to whom an in-depth evaluation in specialized hepatology units must be recommended.

### Supplementary Information

Additional file 1: Supplementary Figure 1. Increased hepatic Bmp8a expression in CCl4 mice. A. Hepatic mRNA levels of Col1a1 and Serpin1 determined by RT-qPCR and normalized to 36b4 gene expression. B. Hepatic mRNA levels of Bmp8a determined by RT-qPCR and normalized to 36b4 gene expression. C and D. Correlation of matched Bmp8a mRNA expression with Col1a1 and Serpin1 mRNA expression respectively. Experimental conditions: mice treated with CCl4 or vehiclei.p. injection twice weekly for 6 weeks.. Data are expressed as fold increase and presented as mean ± SEM relative to control group. *p<0.05, **p<0.01 and ***p<0.005, CCl4 vs. Oil.

## Data Availability

Authors declared that all and the other data supporting the findings of this study are available within the paper. The raw data that support the findings of this study are available from the corresponding author upon reasonable request**.**
