# Supplementary figures and images for: Circulating bone morphogenetic protein 8A is a novel biomarker to predict advanced liver fibrosis
Source: Biomark Res. 2023 Apr 27;11:46. doi: 10.1186/s40364-023-00489-2 (PMC10142503; doi:10.1186/s40364-023-00489-2)

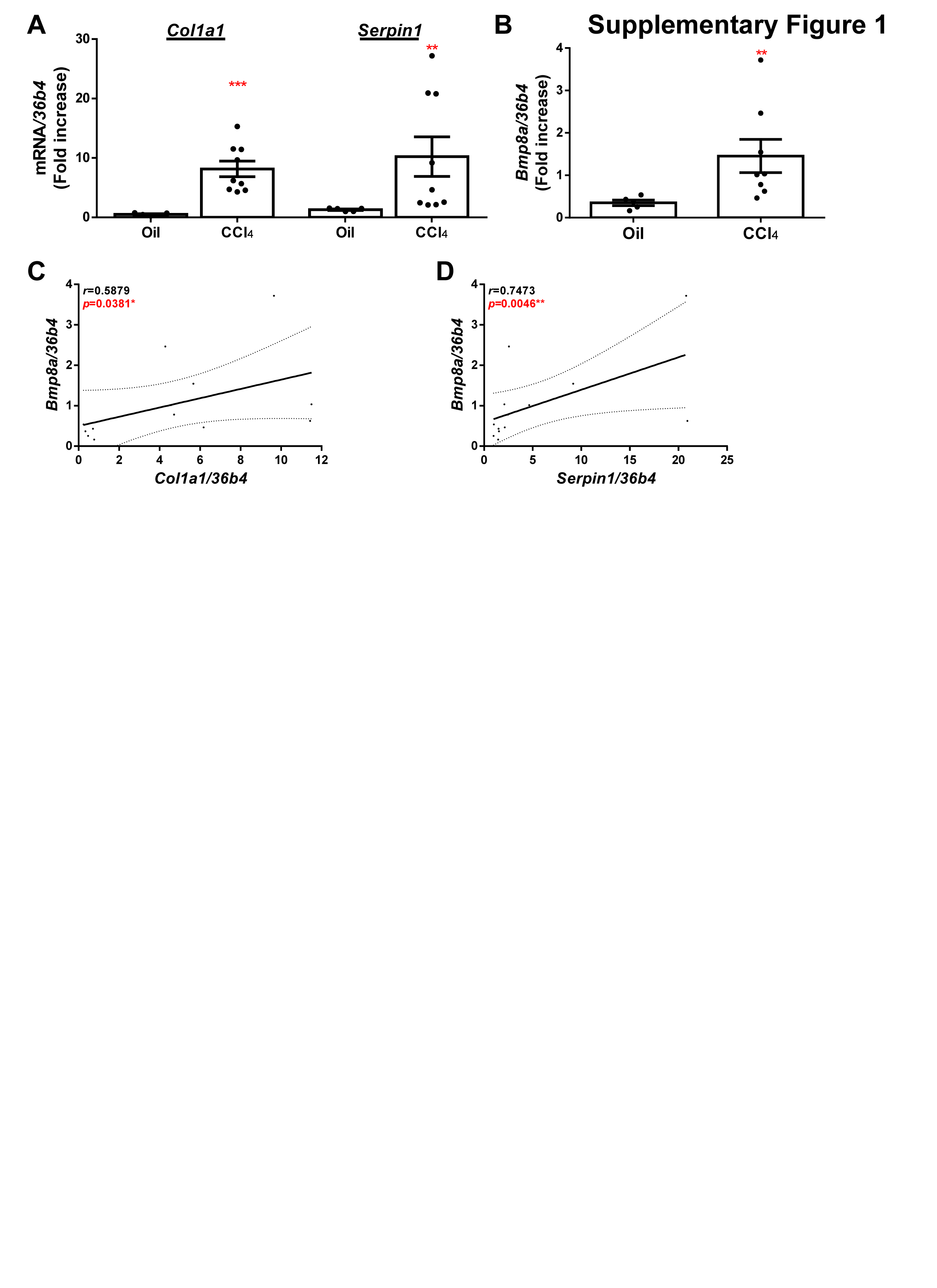

Supplement: Supplementary file 1 — Additional file 1: Supplementary Figure 1. Increased hepatic Bmp8a expression in CCl4 mice. A. Hepatic mRNA levels of Col1a1 and Serpin1 determined by RT-qPCR and normalized to 36b4 gene expression. B. Hepatic mRNA levels of Bmp8a determined by RT-qPCR and normalized to 36b4 gene expression. C and D. Correlation of matched Bmp8a mRNA expression with Col1a1 and Serpin1 mRNA expression respectively. Experimental conditions: mice treated with CCl4 or vehiclei.p. injection twice weekly for 6 weeks.. Data are expressed as fold increase and presented as mean ± SEM relative to control group. *p<0.05, **p<0.01 and ***p<0.005, CCl4 vs. Oil. [file 40364_2023_489_MOESM1_ESM.tif]
